# The Federal ’Right To Try’ Act: An Answer to New Treatments During Terminal Illness?

**Published:** 2017-05-01

**Authors:** Pamela Hallquist Viale

## Abstract

The newly proposed Federal "Right to Try" Act permits terminally ill patients to try experimental therapies that have completed phase I testing but have not been approved by the FDA. However, there are concerning provisions of the legislature regarding liability and costs.

There have been many proposed changes to health care in the past few months, a natural outcome of an election and a change in political perspective. One of the newly proposed laws addresses the right of terminally ill patients to experimental medications. The Federal "Right to Try" Act, H.R. 878, was introduced by Rep. Andy Biggs (R-AZ-5) on February 6, 2017, and follows the pattern of 33 states, which have already passed similar legislature for their residents (Biggs, 2017). The main thrust of the bill calls for "Notwithstanding the Federal Food, Drug, and Cosmetic Act, the Controlled Substances Act, and any other provision of Federal Law, the Federal Government shall not take any action to prohibit or restrict:

(1) The production, manufacture, distribution, prescribing, or dispensing of an experimental drug, biological product, or device that:

- is intended to treat a patient diagnosed with a terminal illness; and
- is authorized by, and in accordance with, State law; and

(2) The possession or use of an experimental drug, biological product, or device

- that is described in subparagraphs (A) and (B) of paragraph (1); and
- for which the patient has received a certification from a physician, who is in good standing with the physician’s certifying organization or board, that the patient has exhausted, or otherwise does not meet qualifying criteria to receive, any other available treatment options."

As advanced practitioners, we take care of patients every day, including those who have no further viable treatment options and must face the prospect of terminal disease. It’s often frustrating and emotional to have the end-of-life conversation with our patients. As providers of care, we naturally want to be able to offer our patients hope for a cure. In the absence of hope, we transition to palliative care and hospice, changing our focus to the management of symptoms and maintaining quality of life. But I’m sure we have all experienced the patient or family who fights end of life with a passion, searching out unproven treatments or experimental therapies. I remember how sad I was to see a good friend (and medical doctor himself) search out antineoplaston therapy, costing him the bulk of his hard-earned savings while he slowly expired of his disease, reeking of urine.

## RAMIFICATIONS OF THE ’RIGHT TO TRY’ ACT

I worry that terminally ill patients will see this potential legislature as a beacon of hope for access to therapies that have not yet been proven effective. The law calls for the experimental drug to have successfully completed a phase I clinical investigation and to remain under investigation in a clinical trial approved by the US Food and Drug Administration (FDA). But as advanced practitioners, we understand the limitations of phase I trials.

These trials usually involve very small numbers of patients and aim at discovering whether people can safely use a new treatment and determining the dose that can be used without causing severe side effects. These trials are not designed to see how well a treatment works. Phase II trials examine the drug’s effectiveness against specific cancers, and phase III trials involve larger groups of patients to determine whether a new treatment is better than standard therapy. These trials are most often randomized to more accurately determine the effectiveness of a new treatment or therapy. They can and usually do take years to accomplish—time a terminally ill patient doesn’t have. This is part of the impetus for the proposed legislature.

## EXPANDED ACCESS

However, terminally ill patients already have an option to try experimental drugs in this country. Patients who have exhausted traditional therapy for their disease can opt for a clinical trial containing new therapies that have not yet been approved, although it is true that patients are usually randomized and may not be selected for the newest therapy arm after all.

The role of the FDA is to review clinical trial data to determine whether the benefits of the new drug outweigh the risk of taking the therapy (Darrow, Sarpatwari, Avorn, & Kesselheim, 2015). This study can take years in the development of a new drug (with the increased involvement of the FDA, drug approval rose, on average, from 2.5 to 8 years; Darrow et al., 2015). Early access may be granted under the FDA’s current national expanded-access program, which states the drug company that makes the treatment agrees to access and the patients will get the treatment for free or at low cost, eliminating the potential for profit-making by the drug company (FDA, 2016). With expanded access, the FDA assesses the condition of the patient (serious or immediately life-threatening), explores whether satisfactory alternative therapies exist, and determines whether access will interfere with pivotal clinical trials. They also balance the benefits vs. potential harm of the request, although for an individual patient, the treating physician need only state that the risk of the disease is greater than the risk of an unproven treatment.

The expanded-access program does allow for patients to receive unproven therapies, without profit to the drug company. Clinical trials offer unproven therapies within the confines of a structured setting, with much of the cost borne by the company sponsoring the trial. The new proposed legislature has three important caveats: drugs that have never been tested on patients before would be included; no liability is awarded (eliminating the ability of the patient or family member to sue the company); and companies can determine the cost of the therapy to the patient, creating the potential for higher drug prices for desperate patients (Zuckerman, 2017).

## FINAL THOUGHTS

I believe the option of clinical trial participation and the ability to apply for the expanded-access program allow patients with terminal illnesses to receive unproven therapies with the protection the FDA currently gives them. I am leery of legislation that dispenses with liability and believe in the power of participation in clinical trials, where results and valuable information regarding side effects can be rigorously collected and evaluated. It is absolutely true that in the past, the FDA has taken an inordinate amount of time to evaluate new therapies that could be used in particular patients who are facing terminal diagnoses; however, evaluation periods and the expanded-access program have improved (Arnst, 2007).

Advanced practitioners should arm themselves with knowledge regarding the proposed bill to answer queries posed by patients facing end of life without viable treatment options. The protection of patients receiving potentially harmful treatments started with the Pure Food and Drug Act of 1906 (Darrow et al., 2015). The FDA currently plays a critical role in this country regarding the safety and appropriate use of drug therapies. I believe further discussion or refinement of the current legislature may be warranted.
